# Importance of *NAB2-STAT6* Fusion in the Diagnosis of Pancreatic Solitary Fibrous Tumor with Hamartoma-Like Features: A Case Report and Review of the Literature

**DOI:** 10.1155/2015/149606

**Published:** 2015-09-06

**Authors:** Kei Tanaka, Takashi Kishimoto, Masayuki Ohtsuka, Yukio Nakatani, Masaru Miyazaki

**Affiliations:** ^1^Department of Surgery, Kensei General Hospital, 604 Kuwata, Sakuragawa, Ibaraki 309-1223, Japan; ^2^General Surgery, Chiba University Graduate School of Medicine, 1-8-1 Inohana, Chuo-ku, Chiba 260-8670, Japan; ^3^Department of Molecular Pathology, Chiba University Graduate School of Medicine, 1-8-1 Inohana, Chuo-ku, Chiba 260-8670, Japan; ^4^Diagnostic Pathology, Chiba University Graduate School of Medicine, 1-8-1 Inohana, Chuo-ku, Chiba 260-8670, Japan

## Abstract

We report a case of pancreatic hamartoma-like solitary fibrous tumor which was differentiated from pancreatic hamartoma with the detection of *NAB2-STAT6* fusion, a specific mutation for solitary fibrous tumors. A pancreatic well-demarcated solid nodule, 21 × 17 mm, of 82-year-old man was surgically enucleated. Microscopic findings were close to a pancreatic hamartoma that consisted of sparsely distributed pancreatic ducts and acini in heavily collagenized fibrous stroma. Neither islet nor peripheral nerve existed in the tumor. The fibroblastic cells in the stroma were immune-positive for CD34, CD99, and bcl-2. But these expressions were not decisive in the differentiation between solitary fibrous tumor and pancreatic hamartoma, because CD34 was positive for both tumors, and CD99 and bcl-2 expressions were not elucidated in the previous cases of pancreatic hamartomas. Thus, we evaluated *NAB2-STAT6* fusion. The fibroblastic cells were positive for STAT6 and sequencing analysis revealed the gene fusion between *NAB2* exon 4 and *STAT6* exon 2, with which the final diagnos is of solitary fibrous tumor was achieved. In conclusion, detection of *NAB2-STAT6* fusion has a great diagnostic value for pancreatic solitary fibrous tumors with hamartoma-like features.

## 1. Introduction

Solitary fibrous tumors (SFTs) are mesenchymal neoplasms, most of which are benign although a part of cases are aggressive with local or distant recurrence. Tumor cells were initially thought to be of mesothelial-cell origin since first report of pleural SFTs was described as localized fibrous mesotheliomas in 1931 [1]. But a series of studies has indicated the fibroblastic or myofibroblastic phenotype of the tumor cells [2–4]. It is now known that SFT can develop in a variety of extrapleural tissues. Among the cases of extrapleural SFT, pancreatic SFT is exceedingly rare; review of the literature yielded 11 prior cases [5–15].

The neoplastic cells in SFTs are spindle or ovoid, and they proliferate in a patternless arrangement characterized by a combination of hyper- and hypocellular areas with varying amounts of collagenized stroma. Hemangiopericytomatous pattern with stag-horn shaped vessels is often observed. Immunohistochemical detection of CD34, CD99, and bcl-2 expressions is commonly employed for the accurate diagnosis of SFT [5]. However, diagnosis is actually challenging in some cases for a wide histological spectrum of this tumor and exceptional immune-phenotype. Recently,* NAB2-STAT6* fusion was discovered in SFTs by means of whole-genome and transcriptome sequencing analysis, and following studies have confirmed that* NAB2-STAT6* fusion is highly sensitive and specific for SFTs [16, 17]. The* NAB2-STAT6* fusion is associated with high nuclear expression of STAT6, so that the STAT6 immunohistochemistry has become a powerful tool in the diagnosis of SFTs [18–20].* NAB2* codes a transcriptional repressor of the early growth response (EGR) zinc-finger transcription factor, which regulates cell differentiation and proliferation. Fusion of activation domain of* STAT6* converts* NAB2* into a transcriptional activator for EGR families and drives tumorigenic effect [16, 21, 22].

Pancreatic hamartoma is a rare benign tumor-like nodule. Microscopically, the distorted epithelial components are distributed in fibrous stroma. Hamartomatous properties are supported by those features of cystic dilation of the ducts, lack of both peripheral nerves and well-formed islets of Langerhans, and lack of concentric elastic fibers in the duct wall. Stromal spindle cells are immune-positive for CD34 as well as those in SFTs [23]. The expressions of CD99 and bcl-2 are unknown, because these expressions were not elucidated in the previously reported pancreatic hamartomas [23–39].

We report a case of pancreatic hamartoma-like SFT. The final diagnosis of SFT was dependent on a validation of* NAB2-STAT6* fusion, because the features were indistinguishable from the pancreatic hamartoma by microscopic examination with conventional immunohistochemical study.

## 2. Case Presentation

An 82-year-old man was referred to our hospital for further evaluation of a pancreatic and a liver nodule that had been found on abdominal CT in the previous hospital. On admission, the patient had no symptoms. PIVKA-2 was elevated (84 mAU/mL), but other tumor markers, that is, CEA, CA19-9, and alpha-fetoprotein, were within normal limits. Abdominal CT revealed a mass, 18 mm in diameter, protruding from the pancreatic tail. The pancreatic mass showed progressive enhancement from the arterial phase to the venous phase on dynamic CT and was hypointense on T2-weighted and diffusion-weighted MR images, which suggested a fibrous tumor. An endoscopic ultrasound with fine-needle aspiration from the pancreatic mass was performed, but paucity of the specimen precluded a diagnosis. At a distance from this nodule, the branch-duct type intraductal papillary-mucinous neoplasm (IPMN) was detected in the preoperative workup. The patient underwent extraction of the pancreatic mass and subsegmentectomy of S8 of the liver. IPMN got a follow-up examination, because it was assessed to be benign on the imaging study. Pathological examination of the surgical specimen of the liver revealed that the liver mass was hepatocellular carcinoma. Eleven months postoperatively, the patient is disease-free and well.

**Figure 1:**
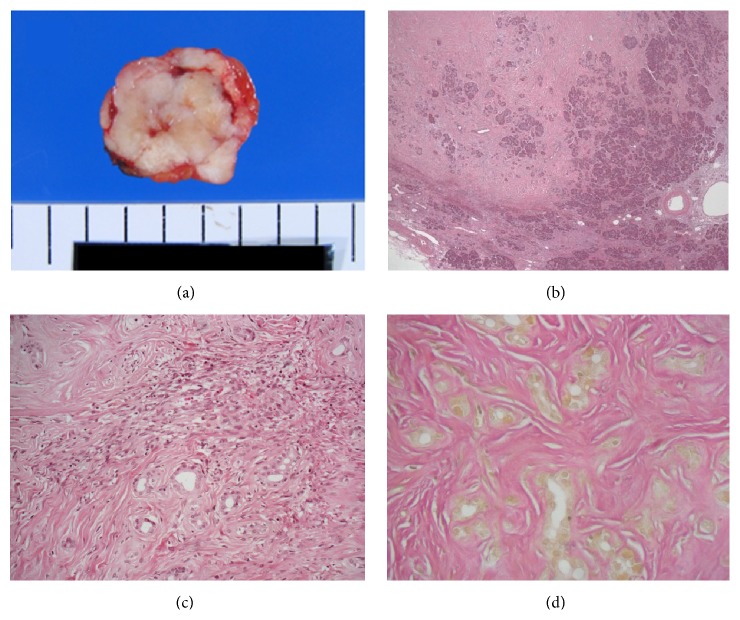
(a) Gross appearance of the surgical specimen showing a well-demarcated and vaguely lobulated ivory-white tumor. (b) Pancreatic parenchyma was haphazardly distributed in the tumor. (c) Spindle cells proliferated in heavily collagenized fibrous stroma. (d) Concentric elastic fibers in the duct walls were not evident with Elastica van Gieson staining.

**Figure 2:**
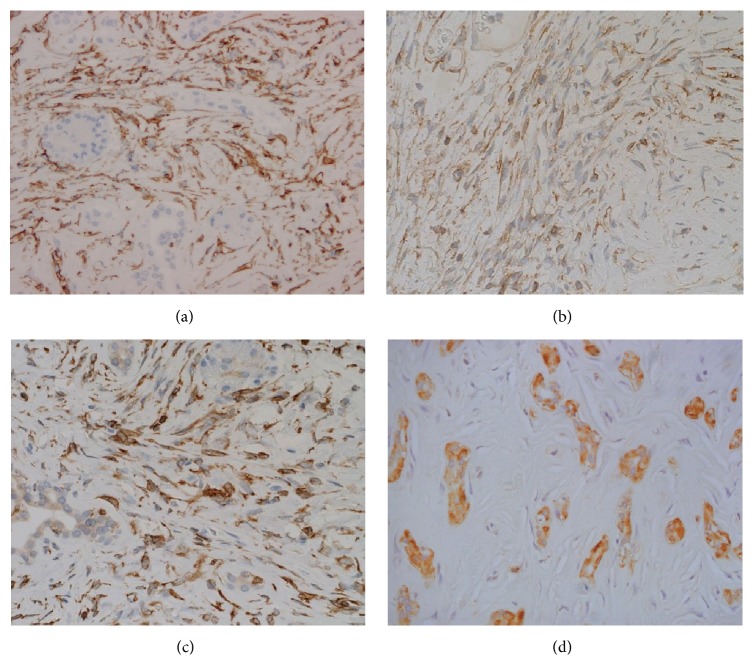
Spindle cells were immune-positive for (a) CD34, (b) CD99, and (c) bcl-2. (d) Ductal epithelia were immune-positive for S-100.

**Figure 3:**
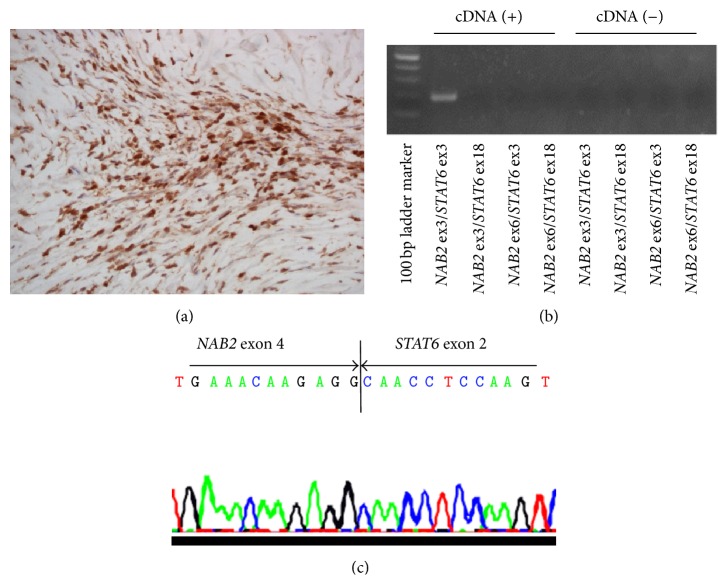
(a) Immunohistochemically, STAT6 was highly expressed in the nuclei of the spindle cells. The results of (b) RT-PCR and (c) sequencing analysis indicated that* NAB2 *exon 4 fused to* STAT6* exon 2.

The specimen resected from the pancreas contained a white, well-demarcated solid nodule, measuring 21 × 17 mm, with a homogeneous appearance on its cut surface (Figure 1(a)). Microscopically, pancreatic acini and ductal tissues were sparsely distributed in the heavily collagenized fibrous stroma with fibroblast-like spindle cells. The cellularity of the spindle cells was low in most areas, but there were a few hypercellular areas where the spindle cells haphazardly proliferated (Figures 1(b) and 1(c)). Mitotic figures, necrosis, and vascular invasion were not identified. Neither islet of Langerhans nor peripheral nerves existed in the whole area. The stroma was hypovascular and the stag-horn-like vessel was not found. Concentric elastic fibers in the duct walls were not evident with Elastica van Gieson staining (Figure 1(d)). Immunohistochemically, the spindle cells were positive for CD34, CD99, and bcl-2 (Figures 2(a)–2(c)) but negative for *α*-SMA, c-kit, DOG1, desmin, S-100, and calretinin. The ductal epithelia were positive for cytokeratin 7 and S-100 protein (Figure 2(d)). These histological and immunohistochemical findings closely resembled pancreatic hamartomas. For the precise diagnosis, we examined the existence of* NAB2-STAT6* fusion in the spindle cells. Immunohistochemical study revealed that STAT6 was highly expressed in the nuclei of the spindle cells (Figure 3(a)). Next, total RNA was extracted from formalin-fixed paraffin embedded tissues with RNeasy FFPE kit (Qiagen, Tokyo, Japan), and RT-PCR was performed to detect* NAB2-STAT6* using two forward primers in* NAB2*, 5′-CAA­GTA­GCC­CGA­GAG­AGC­AC-3′ (exon 3) and 5′-CTG­TGT­GCC­TGC­GAA­GCC-3′ (exon 6), and two reverse primers in* STAT6*, 5′-GGG­AAA­GTC­GAC­ATA­GAG­CC-3′ (exon 3) and 5′-TTC­CAC­GGT­CAT­CTT­GAT­GG-3′ (exon 18) [40]. The condition of PCR was initial denaturing at 95°C for 10 min, followed by 40 cycles of denaturing at 95°C for 30 s, annealing at 55°C for 30 s, and extension at 72°C for 60 s. The primer set of* NAB2* exon 3 and* STAT6* exon 3 was only able to amplify the product (Figure 3(b)). The direct sequence of the product (PRISM 3730 DNA Analyzer, Hokkaido System Science Co., Ltd., Sapporo, Japan) revealed the gene fusion between* NAB2* exon 4 and* STAT6* exon 2 (Figure 3(c)). Based on these results, SFT was finally diagnosed for the pancreatic nodule.

## 3. Discussion

We presented a case of hamartoma-like SFT of the pancreas where the final diagnosis was achieved with* NAB2-STAT6* fusion. Solitary fibrous tumor is one of the mesenchymal neoplasms where pathological diagnosis is difficult because of broad spectrum of histological appearance. For example, cellular variants of SFTs were previously termed hemangiopericytoma as a different histological entity but hemangiopericytoma is now defined as an obsolete synonym in the latest edition of WHO classification [41]. In the SFT in our patient, components of pancreatic parenchyma were haphazardly entrapped in the tumor, which gave rise to a different diagnosis of pancreatic hamartoma. Yamaguchi and colleagues have studied 9 cases of pancreatic hamartomas and noted that distinct characteristic of pancreatic hamartoma is lack of three components: concentric elastic fibers in duct walls, peripheral nerves, and well-formed islets of Langerhans [23]. They also demonstrated the expression of S-100 protein in ductal epithelia in their cases of pancreatic hamartomas [23]. Our presented pancreatic nodule had these characteristics, although the cystic dilated duct that was observed in some previous cases of pancreatic hamartomas was not evident.

We reviewed the previous cases of pancreatic SFTs (11 cases) and pancreatic hamartomas (29 cases) [23–39] and compared histological features of each disease to get knowledge for the differentiation. As per the result summarized in Table 1, some SFTs had hamartoma-like characters resembling our case, such as entrapped acini (6/7, no description in 4 cases) and lack of islet (2/4, no description in 7 cases). On the other hand, several cases of pancreatic hamartomas lack the characteristics of pancreatic hamartomas, although entrapped acini were observed in all cases; cystic dilation of duct was not observed in 5/26 cases (no description in 3 cases), and islets existed in 6/21 cases (no description in 8 cases).

Most of pancreatic SFTs were diagnosed with the immunohistochemical detection of CD34, CD99, and bcl-2 expressions that have been considered valuable for the pathological diagnosis of SFTs. On the other hand, the diagnosis of the pancreatic hamartoma has been mainly achieved by the microscopic appearance. The most examined molecule was CD34, which was examined in 16/29 cases and was positive in 15/16 cases. The expression of bcl-2 was examined only in 5/29 cases and was positive in 2/5 cases. There was no previous case of pancreatic hamartomas in which CD99 expression was examined. Thus, immunohistochemical detection of CD34, CD99, and bcl-2 is inefficient in differentiation of hamartoma-like SFTs from pancreatic hamartomas.

Since Robinson and colleagues discovered that* NAB2-STAT6* gene fusion is the driver mutation of SFTs in 2013, the existence of* NAB2-STAT6* fusion became a diagnostic hallmark of SFTs [16]. The following studies based on this discovery have elucidated the usefulness of immunohistochemical detection of nuclear localization of STAT6 as reliable marker for the diagnosis of SFTs [18–20]. Several variants of* NAB2-STAT6* have been detected; exon 2, 4, 6, or 7 of* NAB2* is fused to exon 2, 3, 5, 6, 17, or 18 of* STAT6* [16, 42]. The presented case had the* NAB2* exon 4-*STAT6* exon 2 fusion. Akaike and colleagues have studied the variation of* NAB2-STAT6* fusion compared with clinicopathologic features and showed that the* NAB2* exon 4-*STAT6 *exon 2 genotype is significantly associated with less aggressive phenotype [42]. Although the existence of* NAB2-STAT6* fusion in pancreatic hamartomas is unknown, proliferation of cells with driver gene mutation highly indicates the neoplastic property. Thus, we advocate the idea that evaluation of* NAB2-STAT6* fusion is most informative in the diagnosis of SFTs and is indispensable for differentiation of hamartoma-like SFTs from true pancreatic hamartomas.

In summary, we described a case of pancreatic SFT with hamartoma-like feature. The evaluation of* NAB2-STAT6* fusion was of great importance in the diagnosis, especially in the differentiation of a pancreatic hamartoma.

## Figures and Tables

**Table 1:** Clinicopathological comparison between solitary fibrous tumors and pancreatic hamartomas.

	SFT (*n* = 11)	Hamartoma (*N* = 29)	Present case
Age (mean)	41–78 (51.8)	0–78 (45.8)	82
Male/female	2/9	15/14	M
Size (cm) (mean)	2.0–18.5 (8.0)	1.0–14 (4.5)	2.1
Cystic change	0/4 (NE 7)	21/26 (NE 3)	(−)
Entrapped acini	6/7 (NE 4)	26/26 (NE 3)	(+)
Lack of islet	2/4 (NE 7)	15/21 (NE 8)	(+)
CD34	11/11	15/16 (NE 13)	(+)
CD99	6/7 (NE 4)	NE 29	(+)
bcl-2	8/8 (NE 3)	2/5 (NE 24)	(+)

SFT: pancreatic solitary fibrous tumor; NE: not examined.
